# Effects of Tannase and Ultrasound Treatment on the Bioactive Compounds and Antioxidant Activity of Green Tea Extract

**DOI:** 10.3390/antiox8090362

**Published:** 2019-09-01

**Authors:** Xiao-Yu Xu, Jin-Ming Meng, Qian-Qian Mao, Ao Shang, Bang-Yan Li, Cai-Ning Zhao, Guo-Yi Tang, Shi-Yu Cao, Xin-Lin Wei, Ren-You Gan, Harold Corke, Hua-Bin Li

**Affiliations:** 1 Guangdong Provincial Key Laboratory of Food, Nutrition and Health, Department of Nutrition, School of Public Health, Sun Yat-Sen University, Guangzhou 510080, China; 2 Department of Food Science and Technology, School of Agriculture and Biology, Shanghai Jiao Tong University, Shanghai 200240, China; 3 Institute of Urban Agriculture, Chinese Academy of Agricultural Sciences, Chengdu 610213, China

**Keywords:** green tea, tannase, ultrasound, antioxidant activity, response surface methodology

## Abstract

The present study investigated the effects of tannase and ultrasound treatment on the bioactive compounds and antioxidant activity of green tea extract. The single-factor experiments and the response surface methodology were conducted to study the effects of parameters on antioxidant activity of green tea extract. The highest antioxidant activity was found under the optimal condition with the buffer solution pH value of 4.62, ultrasonic temperature of 44.12 °C, ultrasonic time of 12.17 min, tannase concentration of 1 mg/mL, and ultrasonic power of 360 W. Furthermore, phenolic profiles of the extracts were identified and quantified by high-performance liquid chromatography. Overall, it was found that tannase led to an increase in gallic acid and a decrease in epigallocatechin gallate, and ultrasounds could also enhance the efficiency of enzymatic reaction.

## 1. Introduction

Reactive oxygen species (ROS) are produced in the human body, and excessive ROS could cause oxidative stress in organisms and promote the development of some chronic diseases [1,2]. The natural antioxidants from plants play an important role in human health. They have been demonstrated to have free radical scavenging activity and protect against oxidative stress [3]. Polyphenols are one of the main natural antioxidants, and have shown many bioactivities and beneficial health effects, such as antioxidant, anti-inflammatory, anticancer, anti-ageing, anti-diabetic, anti-obese, cardioprotective and neuroprotective activities [4,5,6,7,8,9,10]. Other bioactive compounds (such as carotenoids, polysaccharides, alkaloids, and saponins) could also contribute to human health benefits [7,11]. The natural antioxidants could be used for the prevention and management of several diseases, but it has caused some argument. For example, an in vitro antioxidant could exhibit pro-oxidant property in vivo under certain conditions, such as vitamin C [12]. In addition, the antioxidant is usually considered to inhibit the onset of cancers [10]. However, it is also reported that the antioxidant could accelerate lung cancer progression, and increase melanoma metastasis in mice [13,14]. On the other hand, one of the most important uses of natural antioxidants is as the food additives in the food industry [15]. The antioxidants have been defined by several different terms in the literature [16]. For example, when a substance presents at a low concentration compared with that of an oxidizable substrate, it could significantly delay or inhibit the oxidation of that substrate [17]. Many methods have been developed for evaluating the antioxidant property, such as Trolox equivalent antioxidant capacity (TEAC), 2,2-diphenyl-1-picrylhydrazyl (DPPH) radical scavenging, ferric reducing antioxidant power (FRAP), and cellular antioxidant activity assays, and their advantages and disadvantages have been widely reviewed in the literature [18,19,20,21].

Green tea (*Camellia sinensis* L.) is a popular beverage around the world [22,23]. It has been demonstrated that the consumption of green tea is inversely associated with the risk of several chronic diseases, such as cancer, diabetes mellitus, and cardiovascular diseases, and the natural antioxidants are considered as the main contributor to these effects [24,25,26,27]. There have been numerous studies on the antioxidant property of green tea in vitro and in vivo. The consumption of green tea was found to reduce the nicotine-induced toxicity by downregulating the antioxidant- and inflammation-related genes [28]. Besides, the supplement of green tea catechin could decrease the plasma oxidized low-density lipoprotein (LDL) concentration, which probably increased the antioxidant capability of blood and protected the LDL cholesterol from oxidation [29]. Green tea contains various bioactive compounds such as alkaloids, polyphenols, polysaccharides, and L-theanine [30,31,32]. The main alkaloids in green tea are the caffeine, theobromine, and theophylline [33]. The polyphenols in green tea mainly include the catechins, theaflavin, thearubigins, quercetin, and kaempferol mono-, di-, and triglycosides, and some phenolic acids like gallic, coumaric, and caffeic acids [34]. The catechins are mainly composed of (-)-epicatechin (EC), (-)-epigallocatechin (EGC), (-)-epicatechin gallate (ECG), and (-)-epigallocatechin gallate (EGCG) [35]. However, the potential hepatotoxicity induced by the intake of green tea extract has raised a wide concern, and EGCG has been considered as the main contributor of the hepatotoxicity [36,37,38,39]. Thus, the reduction of EGCG in green tea extract, in some cases, could be benefical for the health of human beings [40].

Enzymes have been widely used in the food industry to improve the rate of reaction or obtain the enzymatic hydrolysate [41]. Tannase (tannin acyl hydrolase EC 3.1.1.20) could hydrolyze the ester and depside bonds in gallic acid tannins or hydrolyzable tannins. The treatment of tannase could reduce the contents of several compounds (like EGCG and ECG) in green tea extract by breaking the ester bonds [42]. This leads to a release of gallic acid, which is demonstrated to have high antioxidant activity with health benefits [43]. The increase in the content of gallic acid could greatly improve the antioxidant capacities of the extracts. On the other hand, an ultrasound could accelerate enzymatic reaction [44]. Therefore, the combined treatment of ultrasound and enzyme is indicated to improve the enzymatic reaction with substrates [45]. There are many factors affecting the enzymatic reaction, such as the pH value of buffer solution, the concentration of enzyme, temperature, ultrasonic time, and power [46]. Response surface methodology (RSM) has been used as an effective technique to obtain the optimal process. The main advantage of RSM is that it can reduce the number of experimental trials and require less time than other methods in optimizing a complex process, and the interactive effects and relationships between multiple variables can be analyzed [47,48].

In a previous study, we have found that the green tea Dianqing possessed the strongest antioxidant activity among 30 Chinese teas [49]. The aim of this study was to investigate the effects of several experimental parameters, including the pH value of buffer solution, concentration of tannase, ultrasonic temperature, and ultrasonic time, on the antioxidant activity of green tea extracts, and to optimize the process of tannase and ultrasound treatment by using the single-factor tests and RSM. In addition, this study also compared the efficiency of tannase and ultrasound treatment on improving antioxidant activity of green tea extract. Generally, this study provides a strategy to reduce the content of EGCG in green tea extract, which should have better health benefits, but less hepatotoxicity.

## 2. Materials and Methods

### 2.1. Chemicals and Reagents

Tannase (200 U/g) was purchased from Yuanye Biological Technology Co. Ltd., Shanghai, China. The standard chemicals, including gallic acid, gallocatechin (GC), EGC, catechin, chlorogenic acid, caffeine, EGCG, EC, gallocatechin gallate (GCG), ECG, catechin gallate, ellagic acid, myricetin, quercitrin, and astragalin were purchased from Derick Biotechnology Co. Ltd., Chengdu, China. Formic acid and methanol were of chromatographic grade and obtained from Kermel Chemical Factory, Tianjin, China. The 2,20-azino-bis(3-ethylbenothiazoline-6-sulphonic acid) diammonium salt (ABTS), 6-hydroxy-2,5,7,8-tetramethylchromane-2-carboxylic acid (Trolox), 2,2-diphenyl-1-picrylhydrazyl (DPPH), and 2,4,6-tri(2-pyridyl)-s-triazine (TPTZ) were purchased from Sigma-Aldrich, Saint Louis, MO, USA. All the other chemicals or reagents were of analytical grade. The deionized water was used for all experiments. 

### 2.2. Preparation of Green Tea Extract

Dianqing tea was purchased in the local market of Guangzhou, China, which showed the strongest antioxidant activity among 30 Chinese teas in a previous study [49]. It is a type of green tea produced in Kunming, China. Three different batches of Dianqing tea were bought from different markets, and the mixed tea samples were used for the experiments. The green tea was ground into powders by using a grinder (Royalstar Co. Ltd., Hefei, China), and then the powders were filtered through a 100 meshes sieve. The filtered powders were sealed in plastic bags and stored under 4 °C.

The green tea powders were mixed with 85 °C distilled water (50 g/L, *w*/*v*), and then the mixture was placed in a 85 °C water bath, which was kept at 85 °C for 30 min according to the literature [50,51]. With this tea/water ratio, the tea extract was reported to show stronger antioxidant activity and more abundant phenolic compounds [51]. The mixture was centrifugated at 4200× *g* for 30 min, and the extract was collected and stored at 4 °C for further experiments.

### 2.3. Treatment of Tannase and Ultrasound

Tannase was diluted to different concentrations with 0.1 M citrate–phosphate buffer. The tannase solution was added to the green tea extract. The combined treatment of ultrasound and tannase (UST) was carried out by using an ultrasonic device (Kejin Ultrasonic Equipment Factory, China). The reactions were performed at consistent ultrasonic power of 360 W and other different process parameters in the single-factor tests, including the pH value of citrate–phosphate buffer (3.0, 4.0, 5.0, 6.0, 7.0), tannase concentration (0.0, 0.5, 1.0, 1.5, 2.0, 2.5, 3.0 mg/mL), ultrasonic temperature (25, 35, 45, 55, 65, 75, 85 °C), and ultrasonic time (0, 5, 10, 15, 20, 25, 30, 35 min). After the completion of ultrasonic treatment, the reaction mixture was heated at 100 °C for 10 min to inactivate tannase. The mixture was cooled to room temperature and centrifugated at 4200× *g* for 10 min. The obtained supernatant was stored at −20 °C and further used for the determination of antioxidant activity and HPLC assay. Based on the results of single-factor tests, the pH value of citrate–phosphate buffer, ultrasonic temperature, and time were further optimized by RSM to study their interaction. 

For comparison, the ultrasonic treatment (US) was conducted by adding 0.1 M citrate–phosphate buffer (the optimized pH value) without tannase. The tannase treatment (TAN) was carried out by placing the mixture in the water bath without ultrasound. The green tea extract (GTE) was obtained by dilution of the original extract of green tea with the same buffer solution. The other parameters (such as the pH value, ultrasonic temperature, and time) were the same as those of UST. 

### 2.4. Design by Response Surface Methodology

RSM was used to investigate the impacts of independent variables on the antioxidant capacities (TEAC) of the extracts. Three crucial factors which could significantly affect the antioxidant capacities of the extracts were selected based on the results of single factor tests of UST. Those factors were further used to perform three-variable and five-level central composite design (CCD) via Design Expert v8.0.6 software. Three variables (X_1_, the pH value of citrate–phosphate buffer; X_2_, ultrasonic temperature; X_3_, ultrasonic time) were chosen to be optimized, while other parameters (the concentration of tannase of 1 mg/mL; ultrasonic power of 360 W) were kept constant. 

There was a total of 20 runs of experiments, including 8 combinations of factorial points, 6 combinations of axial points, and 6 replicates of center points. The response values were TEAC values. Data from CCD were analyazed by multiple regressions to fit into the response surface quadratic model, as follows:

Y = β_0_ + ∑β_i_X_i_ + ∑β_ii_ X_i_^2^ + ∑β_ij_ X_i_ X_j_ (1)

where Y was the value of response, X_i_ and X_j_ were different independent variables, β_0_ were the intercept, β_i_, β_ii_, and β_ij_ were the linear, quadratic, and interaction coefficients, respectively.

The analysis of variance (ANOVA) was conducted to analyze the adequacy of the fitted model, of which the significant level was *p* < 0.05. The individual and interactive effects of variables on the response value were visualized on the 3D surface plots. The actual value of the experiment under the optimal conditions was compared with the predicted value, verifying the accuracy of the model.

### 2.5. Determination of Antioxidant Activity

The antioxidant property could be referred to the radicals scavenging capacity, reducing capacity, metal chelating capacity, activity as antioxidative enzyme, and the inhibiting capacity against oxidative enzymes, and many methods have been reported for the evaluation of antioxidant activities [18,19,20,21]. In this study, TEAC, DPPH, and FRAP assays were chosen, because the TEAC and DPPH assays are to determine the radical scavenging capacity, while the FRAP assay is to test the reducing capacity, and they could assess the antioxidant capacities of the extract from different aspects [18]. Furthermore, these methods have been widely used because they are easy to implement and the obtained results are reproducible [52].

#### 2.5.1. Trolox Equivalent Antioxidant Capacity (TEAC) Assay

The antioxidant activities of the extracts were determined by the TEAC assay based on the procedure in the literature [53]. The ABTS^·+^ stock solution was prepared by mixing 7 mmol/L ABTS and 2.45 mmol/L K_2_S_2_O_8_ at the volume ratio of 1:1, and was incubated in a dark place for 16 h at room temperature. The ABTS^·+^ stock solution was diluted to an absorbance of 0.70 ± 0.05 at 734 nm. A 100 μL of diluted extract was mixed with 3.8 mL of ABTS^·+^ working solution, and then the mixture was incubated in a dark place for 6 min at room temperature. The absorbance of the reaction mixture was tested at 734 nm, and the percent of inhibition of absorbance was calculated. Trolox was used as the standard to make the calibration curve. The values of TEAC were presented as μmol Trolox/g dry weight (DW) of green tea.

#### 2.5.2. 2,2-Diphenyl-1-picrylhydrazyl (DPPH) Radical Scavenging Assay

The assay was used to determine the DPPH radical scavenging ability of extracts based on the procedure in the literature, with minor modifications [51]. A 300 μL of diluted extract was mixed with 3 mL of DPPH solution (2 × 10^-4^ M). The reaction mixture was incubated in a dark place for 30 min at room temperature. The control contained the distilled water instead of extracts. The absorbance was determined at 517 nm by using a spectrophotometer. All measurements were carried out in triplicate. The DPPH radical scavenging activity of the extract was caculated as: (1 − As/Ac) × 100%, where As is the absorbance of tested samples and Ac is the absorbance of the control. 

#### 2.5.3. Ferric Reducing Antioxidant Power (FRAP) Assay

The reducing activities of extracts were analyzed according to the literature [49]. The FRAP reaction solution was prepared by mixing 0.3 M acetate buffer (pH 3.6), 0.01 M TPTZ solution, and 0.02 M FeCl_3_ solution at a volume ratio of 10:1:1. A 100 μL of diluted extract was mixed with 3.0 mL FRAP reagent, and the mixture was incubated at room temperature for 4 min. The absorbance was measured at 593 nm. The standard curve was made by using FeSO_4_, and the FRAP values were expressed as μmol Fe^2+^/g DW of green tea.

### 2.6. Phytochemical Analysis of the Extracts by HPLC

The contents of phytochemicals in the extracts were measured by HPLC system, according to the previous method [54]. The HPLC system was equipped with an Agilent Zorbax Extend C18 column (250 × 4.6 mm, 5 μm) and a 2996 photodiode array detector (PDAD) (Waters, Milford, MA, USA). The mobile phase consisted of methanol (solution A) and 0.1% formic acid solution (solution B). The 20 μL sample solution was injected for HPLC analysis. The separation process was conducted with a flow rate at 1.0 mL/min and 35 °C, with the gradient elution procedure as follows: 0 min, 5% (A); 10 min, 20% (A); 15 min, 22% (A); 20 min, 25% (A); 40 min, 40% (A); 50 min, 42% (A); 60 min, 50% (A); 70 min, 95% (A); 70.10 min, 5% (A); 75 min, 5% (A). The phenolic compounds were identified by retention time and UV-vis spectrum compared with those of the standards. The quantification of phenolic compounds was determined by comparing the peak area of the UV response at the maximal absorption wavelength with that observed by mean of pure standards (standard curve method), and was represented as mg/g DW. 

### 2.7. Statistical Analysis

All the experiments were conducted in three independent tests, and data were presented as mean ± standard deviation (SD). The statistical analysis was performed with SPSS 19.0 (IBM SPSS Statistics, IBM Corp, Somers, NY, USA). One-way ANOVA and post hoc least-significant difference (LSD) test were used to determine the significance of differences for each group, with statistical significance at *p* < 0.05.

## 3. Results and Discussions

### 3.1. Results of the Single-Factor Test

#### 3.1.1. Effects of the pH Value of Buffer Solution on Antioxidant Capacities

**Figure 1:**
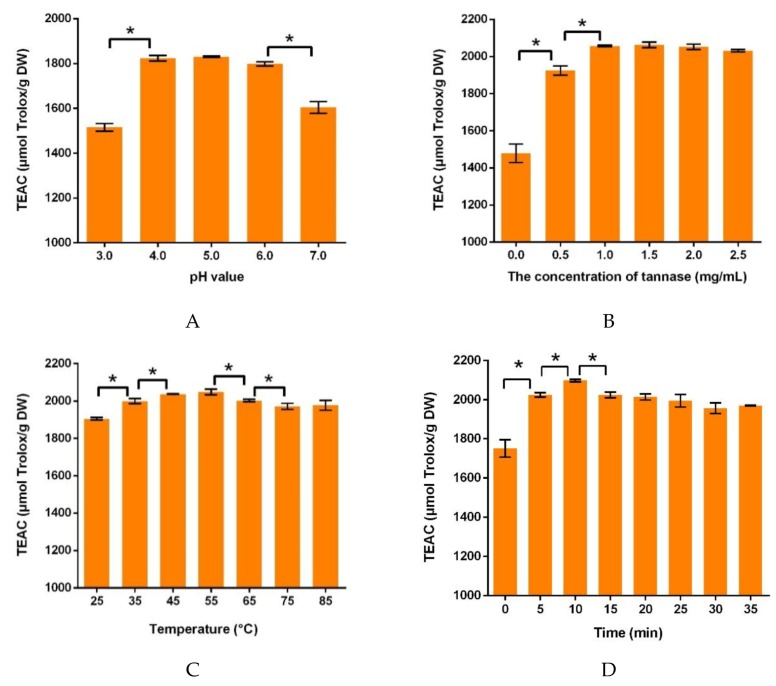
The effects of single factors on the Trolox equivalent antioxidant capacity (TEAC) values. (**A**) the pH value of buffer solution; (**B**) tannase concentration; (**C**) ultrasonic temperature and (**D**) ultrasonic time. DW: dry weight. * Data between groups were significantly different (*p* < 0.05).

The effects of buffer solution pH value on antioxidant capacity of the extracts were investigated in 0.1 M citrate–phosphate buffer. Other parameters were set as the tannase concentration of 1 mg/mL, ultrasonic temperature of 35 °C, and ultrasonic time of 30 min. Figure 1A showed that the TEAC value increased at pH value of 3.0 to 5.0, and decreased at pH value of 5.0 to 7.0. It showed that tannase had an optimal pH value of 5.0, and the result is similar to a study reported by Costa et.al, which illustrated that the free tannase was active and stable at pH 5.0 [55]. The pH value of buffer solution is one of the most important parameters affecting enzymatic activity. It was mainly related to the protonation and deprotonation of amino acids at the active site, and the alternation in conformation induced by the ionization from other amino acids [56]. Thus, using the buffer at an appropriate pH value could greatly enhance the activity of enzymes. Tannase was mainly produced by fungus, and the fungal tannase was generally acidic protein. The activity of tannase increased at the acidic solution and began to decrease when the solution became alkaline [57]. There were also other studies resulting in the pH value around 5.0 as the optimum for tannase activity [58]. Thus, the pH value of 5.0 was chosen for the following experiments.

#### 3.1.2. Effects of Tannase Concentration on Antioxidant Capacities

The tannase concentrations ranged from 0.0 to 2.5 mg/mL, which were evaluated as its influence on the antioxidant capacities of the extracts, while other conditions were controlled as 0.1 M citrate–phosphate buffer (pH 5.0), ultrasonic temperature of 35 °C, and ultrasonic time of 30 min. In addition, 50 g/L of the tea/water ratio was utilized, because the tea extract was reported to show stronger antioxidant activity and more abundant phenolic compounds with this ratio [51]. The results in Figure 1B revealed that the antioxidant capacities initially increased in the range of 0.0–1.0 mg/mL. As the concentration of tannase was subsequently increased, there was no significant change in TEAC values. When the concentration of substrate was constant and the amount of enzyme was small, substrate was not saturated by the enzyme [59]. With more enzyme added to substrate, there was a greater enzymatic reaction. However, the substrate would be saturated by a certain amount of enzyme, and the effect of enzyme was no longer significant on the substrate. The adding of tannase enhanced the biotransformation of tea polyphenols, and more antioxidant compounds were produced from green tea extracts. Taking the cost and effectiveness into consideration, 1 mg/mL was selected as the concentration of tannase in following experiments, because TEAC values had little difference when tannase concentration was beyond 1 mg/mL. 

#### 3.1.3. Effects of Ultrasonic Temperature on Antioxidant Capacities

To investigate the effect of temperature in ultrasound treatment on the antioxidant capacities, the temperature was varied from 25 to 85 °C, and other parameters were set as 0.1 M citrate–phosphate buffer (pH 5.0), tannase concentration of 1 mg/mL, and ultrasonic time of 30 min. As shown in Figure 1C, the TEAC value increased under the ultrasonic temperature from 25 to 45 °C. The antioxidant values at the temperature of 45 and 55 °C made no significant difference. With a further increase in temperature from 55 to 85 °C, there was a downward trend in the antioxidant capacity because the high temperature could decrease the activity of enzyme [60]. Thus, 45 °C was selected in subsequent experiments.

#### 3.1.4. Effects of Ultrasonic Time on Antioxidant Capacities

The antioxidant capacity of the extracts varied greatly depending on the time in ultrasound treatment. The sample was treated under different ultrasonic time, and other procedures were 0.1 M citrate–phosphate buffer (pH 5.0), tannase concentration of 1 mg/mL, and ultrasonic temperature of 45 °C. As shown in Figure 1D, the antioxidant capacities increased significantly from 0 to 10 min, and it reached the peak at 10 min. The prolongation of ultrasonic time allowed enzymes to react with substrate completely, which led to an increase in the antioxidant capacities. However, after the ultrasonic time was longer than 10 min, the antioxidant capacities began to decline. Due to the too-long ultrasonic time, the activity of enzyme could decrease, and antioxidants could be degraded. Therefore, the time of 10 min was selected in the treatment.

### 3.2. Results by Response Surface Methodology

#### 3.2.1. Statistical Analysis and Model Fitting

Based on the results of single-factor tests, three individual factors (the pH value of buffer solution, ultrasonic temperature and ultrasonic time) were further optimized via CCD. The actual and coded levels of three individual variables and corresponding response values are listed in Table 1. A mathematical model was developed to evaluate the relationship between variables, in which the TEAC values were presented as a function of independent variables in selected ranges.

Y = 2036.55 − 53.49 X_1_ − 10.25 X_2_ + 30.81 X_3_ − 23.82 X_1_ X_2_ + 2.82 X_1_ X_3_ − 18.48 X_2_ X_3_ − 66.74 X_1_^2^ − 52.69 X_2_^2^ − 36.24 X_3_^2^ (2)

where X_1_, X_2_, and X_3_ are the pH value of buffer solution, ultrasonic temperature, and ultrasonic time, respectively. Y represents the TEAC values. 

The ANOVA of the response surface quadratic model is shown in Table 2. The p-value was used to evaluate the statistical significance of the regression model. In general, the model can be considered as significant if the p-value is smaller than 0.05. As shown in Table 2, the regression model was indicated to be significant (*p* < 0.05). In addition, the linear coefficients (X_1_, X_3_) and quadratic coefficients (X_1_^2^, X_2_^2^, X_3_^2^) had statistical significance (*p* < 0.05). Furthermore, the lack-of-fit testing was non-significant (*p* = 0.1508), which further verified that the model was suitable in predicting the response. 

After the response surface regression, the determination coefficient value (*R^2^*) of the predicted model was 0.9450, which suggested a good fit of the model. Also, 94.50% of the variation could be reflected by the predicted model. The adjusted *R^2^* was 0.8954 which was closed to *R^2^*, suggesting that the actual responses were highly correlated with the predicted responses. Moreover, the coefficient of variation (CV) represented the reproducibility of a model. The CV was 1.61%, which was smaller than 10%, indicating that the model was reproducible.

#### 3.2.2. Response Surface Plots and Graphical Analysis

**Figure 2:**
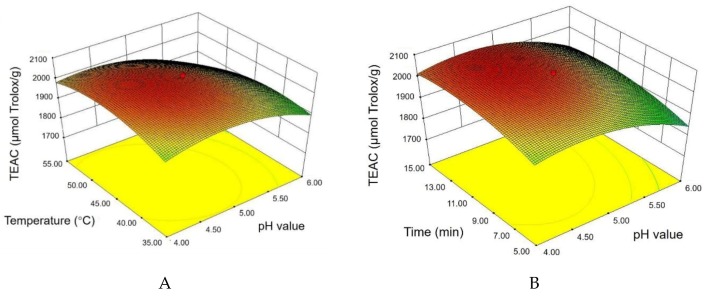

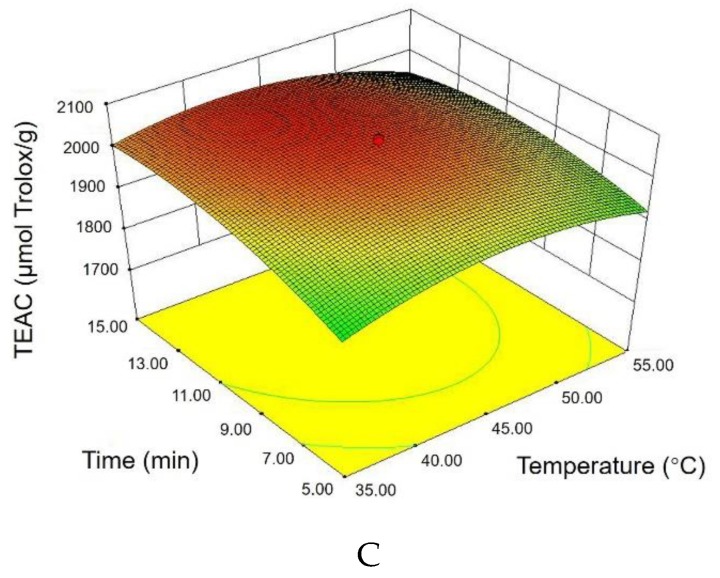
Graphical analysis of interactive effects on TEAC values. (**A**) the pH value of buffer solution and ultrasonic temperature; (**B**) the pH value of buffer solution and ultrasonic time; (**C**) ultrasonic temperature and time.

The interrelation of independent variables and response values were visually displayed via the 3D response plots in Figure 2. As is shown in Figure 2A, the TEAC values were performed as a function of pH value and ultrasonic temperature with a fixed ultrasonic time. When ultrasonic time was kept at 10 min, the TEAC values increased slowly with the increase of pH value from 4.00 to 5.00, then dropped from 5.00 to 6.00. In addition, the TEAC values increased with the increase of ultrasonic temperature from 35 °C to 45 °C, then decreased slightly from 45 °C to 55 °C. Figure 2B presents the interactions between the pH value and ultrasonic time on the TEAC values at 45 °C. The effects of the pH value on the TEAC value were similar to that shown in Figure 2A. The increase in the ultrasonic time slightly elevated the TEAC values from 5.00 to 15.00 min. Moreover, Figure 2C displays that under the condition of fixed pH value, the antioxidant capacities markedly improved as the ultrasonic temperature and time increased. The effect of ultrasonic time was more potent than that of ultrasonic temperature. According to the results of ANOVA and response surfaces plots, the pH value and ultrasonic time had stronger effects on antioxidant capacities than that of ultrasonic temperature.

#### 3.2.3. Optimal Treatment Conditions

Based on the single-factor tests and RSM study, the optimal condition for tannase and ultrasound treatment of the extracts was 0.1 M citrate–phosphate buffer with a pH value of 4.62, ultrasonic temperature of 44.11 °C, ultrasonic time of 12.17 min, tannase concentration of 1 mg/mL, and ultrasonic power of 360 W. The determination of antioxidant capacities was conducted three times under the optimal conditions, and TEAC value was 2028.66 ± 4.59 μmol Trolox/g DW, which was close to the predicted values of 2053.73 μmol Trolox/g DW.

### 3.3. Comparison of Different Treatments

**Figure 3:**
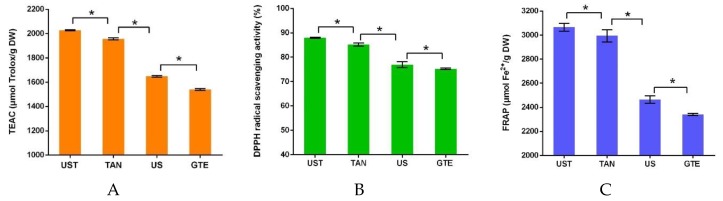
The comparison of antioxidant capacities of the extracts with different treatments. * Data between groups were significantly different (*p* < 0.05).

The effects of different treatments were compared on the antioxidant capacities of the extracts (Figure 3A). The results showed that US had higher antioxidant capacities than that of GTE, and TAN exhibited a significant increase in antioxidant capacities compared with GTE. Furthermore, UST showed higher antioxidant capacities than that of TAN. That is, the tannase could induce biotransformation in polyphenols and produced a large number of antioxidant compounds like gallic acids, and ultrasound could improve the efficiency of an enzymatic reaction. In addition, the results obtained by the DPPH and FRAP assays showed the similar trends as that by TEAC assay (Figure 3B and Figure 3C).

### 3.4. Antioxidants of the Extracts

**Figure 4:**
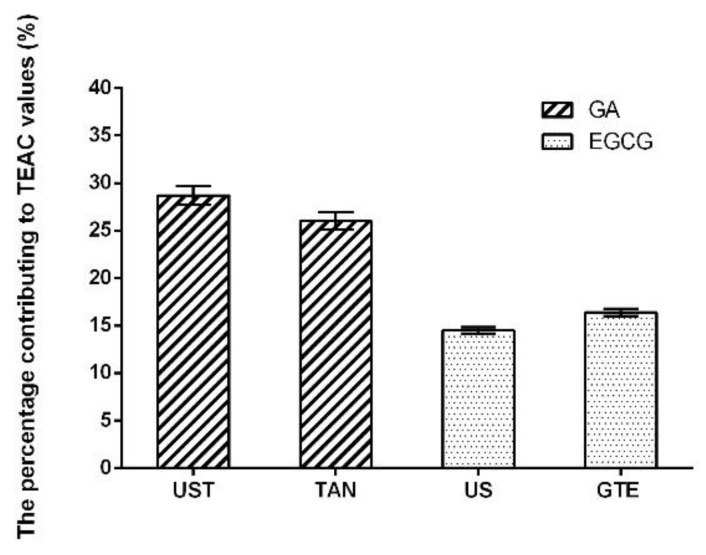
The percentage of gallic acid (GA) and epigallocatechin gallate (EGCG) contributing to the TEAC values of extracts for different treatments.

The antioxidants in the extracts obtained from different treatments were identified and quantified by HPLC. Ten antioxidants were identified and quantified, including gallic acid, GC, GCG, EC, EGC, ECG, EGCG, caffeine, ellagic acid, and astragalin (Table 3). The addition of tannase in green tea extract resulted in a significant increase in gallic acid, EGC, and EC, and a significant reduction in EGCG, ECG, and GCG (*p* < 0.05). By using the pure standards as the reference, the results revealed that the antioxidant capacities of UST and TAN were mainly attributed to the content of gallic acid, which took the percentage of 28.70% and 26.00% in the TEAC values, respectively. On the other hand, the antioxidant capacities of US and GTE mainly depended on the contents of EGCG, with the percentage of 14.5% and 16.38% in the TEAC values, respectively (Figure 4). It confirmed that tannase catalyzes the hydrolysis of ester and depside bonds in gallic acid tannins or hydrolyzable tannins. In addition, the combined treatment of tannase and ultrasound significantly increased the yield of gallic acid in the extracts compared with those only treated with tannase (*p* < 0.05). It indicated that the simultaneous treatment of tannase and ultrasound not only accelerated the release of phenolic compounds from green tea extracts, but also increased the rate of enzymatic reaction on biotransformation, resulting in the improved antioxidant activity of green tea extracts. Furthermore, other compounds like caffeine, ellagic acid, and astragalin were changed slightly with the use of ultrasound or tannase. 

## 4. Conclusions

The effects of tannase and ultrasound treatment have been studied on the bioactive compounds and antioxidant activity of green tea extract. The optimal conditions were 0.1 M citrate–phosphate buffer at pH value of 4.62, ultrasonic temperature of 44.12 °C, ultrasonic time of 12.17 min, tannase concentration of 1 mg/mL, and ultrasonic power of 360 W. The tannase enzymatic reaction led to bioconversion of bioactive components in green tea extracts, and resulted in the increase of the antioxidant activity. The ultrasound exerted positive effects on the tannase enzymatic reaction, and the combined treatment of tannase and ultrasound markedly increased the antioxidant activity of the green tea extract. Furthermore, the ten antioxidants in the extracts have been identified and quantified by an HPLC method. The results showed an increase in gallic acid, EC, and EGC, and a decrease in EGCG, ECG, and GCG after the treatment. Since the consumption of EGCG has been reported to have potential toxicity in liver, the reduction of EGCG content via the tannase and ultrasound treatment might improve the human benefits of green tea extracts. The simultaneous ultrasound and tannase treatment could be a potent tool in the food industry with low cost, high efficiency, and shortened processing steps. It also could promote the production of natural antioxidants which may be used as nutritional supplements or food additives in the food industry. In the future, the in vivo antioxidant activities of these extracts should be evaluated in order to compare the effects of different treatments on the in vivo and in vitro antioxidant activites. In addition, more tea samples could be tested to verify the effectivity of these methods, such as white, yellow, oolong, black, and dark teas.

## Figures and Tables

**Table 1:** Central composite design and result of response surface method analysis.

Run	pH (X_1_)	Temperature (X_2_, °C)	Time (X_3_, min)	TEAC Value (Y, μmol Trolox/g DW)	
				Actual Value	Predicted Value
1	6 (1)	35 (−1)	5 (−1)	1798.00	1809.36
2	5 (0)	45 (0)	10 (0)	2001.15	2036.55
3	5 (0)	45 (0)	18.41 (1.68)	1993.44	1985.88
4	6 (1)	35 (−1)	15 (1)	1930.55	1913.58
5	4 (−1)	35 (−1)	5 (−1)	1871.32	1874.33
6	5 (0)	45 (0)	10 (0)	2028.64	2036.55
7	5 (0)	45 (0)	10 (0)	2056.13	2036.55
8	4 (−1)	55 (1)	5 (−1)	1895.76	1938.43
9	3.32 (−1.68)	45 (0)	10 (0)	1969.07	1937.75
10	4 (−1)	35 (−1)	15 (1)	1953.80	1967.27
11	5 (0)	45 (0)	10 (0)	2057.66	2036.55
12	5 (0)	28.18 (−1.68)	10 (0)	1898.81	1904.75
13	5 (0)	61.82 (1.68)	10 (0)	1912.56	1870.27
14	4 (−1)	55 (1)	15 (1)	1943.11	1957.46
15	5 (0)	45 (0)	10 (0)	2019.47	2036.55
16	5 (0)	45 (0)	1.59 (−1.68)	1911.03	1882.23
17	6 (1)	55 (1)	5 (−1)	1765.93	1778.17
18	6.68 (1.68)	45 (0)	10 (0)	1762.88	1757.84
19	5 (0)	45 (0)	10 (0)	2050.02	2036.55
20	6 (1)	55 (1)	15 (1)	1785.79	1808.48

**Table 2:** Analysis of variance (ANOVA) of the fitted polynomial quadratic model.

Source	Sum of Squares	Df	Mean Square	F Value	P-Value	Significant
Model	165,385.81	9	18,376.20	19.08	< 0.0001	Significant
X_1_-pH	39,072.88	1	39,072.88	40.56	< 0.0001	
X_2_-Temperautre	1434.63	1	1434.63	1.49	0.2503	
X_3_-Time	12,967.00	1	12,967.00	13.46	0.0043	
X_1_ X_2_	4540.31	1	4540.31	4.71	0.0551	
X_1_ X_3_	63.69	1	63.69	0.066	0.8023	
X_2_ X_3_	2731.27	1	2731.27	2.84	0.1231	
X_1_^2^	64,181.85	1	64,181.85	66.62	< 0.0001	
X_2_^2^	40,016.60	1	40,016.60	41.54	< 0.0001	
X_3_^2^	18,924.01	1	18,924.01	19.64	0.0013	
Residual	9633.42	10	963.34			
Lack of Fit	7021.77	5	1404.35	2.69	0.1508	not significant
Pure Error	2611.66	5	522.33			
Cor Total	175,019.24	19				

*R*^2^ = 0.9450, Adj *R*^2^ = 0.8954, CV% = 1.61.

**Table 3:** The contents of antioxidants in green tea extracts with different treatments (mg/g DW).

Antioxidants	UST	TAN	US	GTE
Gallic acid	31.83 ± 0.74 ^a^	27.82 ± 0.47 ^b^	2.16 ± 0.05 ^c^	1.97 ± 0.03 ^c^
Gallocatechin	0.32 ±0.09	0.32 ± 0.02	0.21 ± 0.07	0.21 ± 0.01
Epicatechin	1.96 ± 0.31 ^a^	2.12 ± 0.17 ^a^	0.70 ± 0.06 ^b^	0.71 ± 0.02 ^b^
Epigallocatechin	2.02 ± 0.73 ^a^	2.07 ± 0.22 ^a^	0.29 ± 0.02 ^b^	0.30 ± 0.01 ^b^
Epigallocatechin gallate	0.16 ± 0.06 ^c^	0.16 ± 0.10 ^c^	11.55 ± 0.14 ^b^	12.18 ± 0.20 ^a^
Epicatechin gallate	0.16 ± 0.02 ^b^	0.19 ± 0.04 ^b^	6.33 ± 0.46 ^a^	6.03 ± 0.60 ^a^
Gallocatechin gallate	0.17 ± 0.11 ^b^	0.17 ± 0.08 ^b^	0.71 ± 0.02 ^a^	0.58 ± 0.09 ^a^
Caffeine	17.00 ± 0.63 ^a,b^	17.59 ± 0.38 ^a^	16.60 ± 0.17 ^b^	16.39 ± 0.32 ^b^
Ellagic acid	1.31 ± 0.04	1.32 ± 0.55	1.41 ± 0.09	0.93 ± 0.19
Astragalin	0.58 ± 0.02 ^a^	0.45 ± 0.10 ^b^	0.53 ± 0.04 ^a,b^	0.51 ± 0.02 ^a,b^

^a, b, c^ Data between groups were significantly different (*p* < 0.05).

## References

[1] Ende W.V.D., Peshev D., De Gara L. (2011). Disease prevention by natural antioxidants and prebiotics acting as ROS scavengers in the gastrointestinal tract. Trends Food Sci. Technol..

[2] Nuutila A.M., Puupponen-Pimiä R., Aarni M., Oksman-Caldentey K.-M. (2003). Comparison of antioxidant activities of onion and garlic extracts by inhibition of lipid peroxidation and radical scavenging activity. Food Chem..

[3] Paran E., Novack V., Engelhard Y.N., Hazan-Halevy I. (2009). The effects of natural antioxidants from tomato extract in treated but uncontrolled hypertensive patients. Cardiovasc. Drugs Ther..

[4] Pandey K.B., Rizvi S.I. (2009). Plant polyphenols as dietary antioxidants in human health and disease. Oxidative Med. Cell. Longev..

[5] Li A.-N., Li S., Zhang Y.-J., Xu X.-R., Chen Y.-M., Li H.-B. (2014). Resources and Biological Activities of Natural Polyphenols. Nutrients.

[6] He F.J., Nowson C.A., Lucas M., MacGregor G.A. (2007). Increased consumption of fruit and vegetables is related to a reduced risk of coronary heart disease: Meta-analysis of cohort studies. J. Hum. Hypertens..

[7] Zhang Y.-J., Gan R.-Y., Li S., Zhou Y., Li A.-N., Xu D.-P., Li H.-B. (2015). Antioxidant Phytochemicals for the Prevention and Treatment of Chronic Diseases. Molecules.

[8] Dauchet L., Amouyel P., Hercberg S., Dallongeville J. (2006). Fruit and vegetable consumption and risk of coronary heart disease: A meta-analysis of cohort studies. J. Nutr..

[9] Shen Y., Zhang H., Cheng L., Wang L., Qian H., Qi X. (2016). In vitro and in vivo antioxidant activity of polyphenols extracted from black highland barley. Food Chem..

[10] Zhou Y., Zheng J., Li Y., Xu D.-P., Li S., Chen Y.-M., Li H.-B. (2016). Natural Polyphenols for Prevention and Treatment of Cancer. Nutrients.

[11] Zhou Y., Li Y., Zhou T., Zheng J., Li S., Li H.-B. (2016). Dietary Natural Products for Prevention and Treatment of Liver Cancer. Nutrients.

[12] Podmore I.D., Griffiths H.R., Herbert K.E., Mistry N., Mistry P., Lunec J. (1998). Vitamin C exhibits pro-oxidant properties. Nature.

[13] Sayin V.I., Ibrahim M.X., Larsson E., Nilsson J.A., Lindahl P., Bergo M.O. (2014). Antioxidants Accelerate Lung Cancer Progression in Mice. Sci. Transl. Med..

[14] Le Gal K., Ibrahim M.X., Wiel C., Sayin V.I., Akula M.K., Karlsson C., Dalin M.G., Akyürek L.M., Lindahl P., Nilsson J. (2015). Antioxidants can increase melanoma metastasis in mice. Sci. Transl. Med..

[15] Caleja C., Barros L., Antonio A.L., Oliveira M.B.P., Ferreira I.C. (2017). A comparative study between natural and synthetic antioxidants: Evaluation of their performance after incorporation into biscuits. Food Chem..

[16] Carocho M., Ferreira I.C. (2013). A review on antioxidants, prooxidants and related controversy: Natural and synthetic compounds, screening and analysis methodologies and future perspectives. Food Chem. Toxicol..

[17] Halliwell B., Gutteridge J.M. (1995). The definition and measurement of antioxidants in biological systems. Free. Radic. Biol. Med..

[18] Xu D.-P., Li Y., Meng X., Zhou T., Zhou Y., Zheng J., Zhang J.-J., Li H.-B. (2017). Natural Antioxidants in Foods and Medicinal Plants: Extraction, Assessment and Resources. Int. J. Mol. Sci..

[19] Apak R., Özyürek M., Güçlü K., Çapanoğlu E. (2016). Antioxidant Activity/Capacity Measurement. 1. Classification, Physicochemical Principles, Mechanisms, and Electron Transfer (ET)-Based Assays. J. Agric. Food Chem..

[20] Apak R., Özyürek M., Güçlü K., Çapanoğlu E. (2016). Antioxidant Activity/Capacity Measurement. 2. Hydrogen Atom Transfer (HAT)-Based, Mixed-Mode (Electron Transfer (ET)/HAT), and Lipid Peroxidation Assays. J. Agric. Food Chem..

[21] Apak R., Özyürek M., Güçlü K., Çapanoğlu E. (2016). Antioxidant Activity/Capacity Measurement. 3. Reactive Oxygen and Nitrogen Species (ROS/RNS) Scavenging Assays, Oxidative Stress Biomarkers, and Chromatographic/Chemometric Assays. J. Agric. Food Chem..

[22] Xiao J., Huo J., Jiang H., Yang F. (2011). Chemical compositions and bioactivities of crude polysaccharides from tea leaves beyond their useful date. Int. J. Biol. Macromol..

[23] Higdon J.V., Frei B. (2003). Tea catechins and polyphenols: Health effects, metabolism, and antioxidant functions. Crit. Rev. Food Sci. Nutr..

[24] Nagao T., Hase T., Tokimitsu I. (2007). A Green Tea Extract High in Catechins Reduces Body Fat and Cardiovascular Risks in Humans. Obesity.

[25] Xu X.-Y., Zhao C.-N., Cao S.-Y., Tang G.-Y., Gan R.-Y., Li H.-B. (2019). Effects and mechanisms of tea for the prevention and management of cancers: An updated review. Crit. Rev. Food Sci. Nutr..

[26] Cao S.Y., Zhao C.N., Gan R.Y., Xu X.Y., Wei X.L., Corke H., Atanasov A.G., Li H.B. (2019). Effects and mechanisms of tea and its bioactive compounds for the prevention and treatment of cardiovascular diseases: An updated review. Antioxidants.

[27] Meng J.M., Cao S.Y., Wei X.L., Gan R.Y., Wang Y.F., Cai S.X., Xu X.Y., Zhang P.Z., Li H.B. (2019). Effects and mechanisms of tea for the prevention and management of diabetes mellitus and diabetic complications: An updated review. Antioxidants.

[28] Al-Awaida W.J., Zihlif M.A., Al-Ameer H.J., Sharab A., Akash M., Aburubaiha Z.A., Fattash I.A., Imraish A., Ali K.H. (2019). The effect of green tea consumption on the expression of antioxidant- and inflammation-related genes induced by nicotine. J. Food Biochem..

[29] Inami S., Takano M., Yamamoto M., Murakami D., Tajika K., Yodogawa K., Yokoyama S., Ohno N., Ohba T., Sano J. (2007). Tea catechin consumption reduces circulating oxidized low-density lipoprotein. Int. Heart J..

[30] Conde V.R., Alves M.G., Oliveira P.F., Silva B.M. (2015). Tea (Camellia sinensis (L.)): A Putative anticancer agent in bladder carcinoma?. Anti-Cancer Agents Med. Chem..

[31] Gan R.Y., Li H.B., Sui Z.Q., Corke H. (2018). Absorption, metabolism, anti-cancer effect and molecular targets of epigallocatechin gallate (EGCG): An updated review. Crit. Rev. Food Sci. Nutr..

[32] Cavet M.E., Harrington K.L., Vollmer T.R., Ward K.W., Zhang J.-Z. (2011). Anti-inflammatory and anti-oxidative effects of the green tea polyphenol epigallocatechin gallate in human corneal epithelial cells. Mol. Vis..

[33] Del Rio D., Stewart A.J., Mullen W., Burns J., Lean M.E.J., Brighenti F., Crozier A. (2004). HPLC-MSnAnalysis of Phenolic Compounds and Purine Alkaloids in Green and Black Tea. J. Agric. Food Chem..

[34] Rusak G., Komes D., Likić S., Horžić D., Kovač M. (2008). Phenolic content and antioxidative capacity of green and white tea extracts depending on extraction conditions and the solvent used. Food Chem..

[35] Peluso I., Serafini M. (2017). Antioxidants from black and green tea: From dietary modulation of oxidative stress to pharmacological mechanisms. Br. J. Pharmacol..

[36] Isomura T., Suzuki S., Origasa H., Hosono A., Suzuki M., Sawada T., Terao S., Muto Y., Koga T. (2016). Liver-related safety assessment of green tea extracts in humans: A systematic review of randomized controlled trials. Eur. J. Clin. Nutr..

[37] Dostal A.M., Samavat H., Bedell S., Torkelson C., Wang R., Swenson K., Le C., Wu A.H., Ursin G., Yuan J.-M. (2015). The safety of green tea extract supplementation in postmenopausal women at risk for breast cancer: Results of the Minnesota Green Tea Trial. Food Chem. Toxicol..

[38] Bedrood Z., Rameshrad M., Hosseinzadeh H. (2018). Toxicological effects of *Camellia sinensis* (green tea): A review. Phytother. Res..

[39] Mazzanti G., Di Sotto A., Vitalone A. (2015). Hepatotoxicity of green tea: An update. Arch. Toxicol..

[40] Hu J., Webster D., Cao J., Shao A. (2018). The safety of green tea and green tea extract consumption in adults —Results of a systematic review. Regul. Toxicol. Pharmacol..

[41] Toushik S.H., Lee K., Kim K. (2017). Functional Applications of Lignocellulolytic Enzymes in the Fruit and Vegetable Processing Industries. J. Food Sci..

[42] Roberto B.S., Macedo G.A., Macedo J.A., Martins I.M., Nakajima V.M., Allwood J.W., Stewart D., McDougall G.J. (2016). Immobilized tannase treatment alters polyphenolic composition in teas and their potential anti-obesity and hypoglycemic activities in vitro. Food Funct..

[43] Yen G.-C., Duh P.-D., Tsai H.-L. (2002). Antioxidant and pro-oxidant properties of ascorbic acid and gallic acid. Food Chem..

[44] Chemat F., Huma Z.E., Khan M.K. (2011). Applications of ultrasound in food technology: Processing, preservation and extraction. Ultrason. Sonochem..

[45] Soares A.D.S., Augusto P.E.D., Júnior B.R.D.C.L., Nogueira C.A., Vieira É.N.R., De Barros F.A.R., Stringheta P.C., Ramos A.M. (2019). Ultrasound assisted enzymatic hydrolysis of sucrose catalyzed by invertase: Investigation on substrate, enzyme and kinetics parameters. LWT.

[46] Li A.-N., Li S., Li Y., Xu D.-P., Li H.-B. (2016). Optimization of Ultrasound-Assisted Extraction of Natural Antioxidants from the Osmanthus fragrans Flower. Molecules.

[47] Yolmeh M., Najafi M.B.H., Farhoosh R. (2014). Optimisation of ultrasound-assisted extraction of natural pigment from annatto seeds by response surface methodology (RSM). Food Chem..

[48] Ye C.-L., Jiang C.-J. (2011). Optimization of extraction process of crude polysaccharides from Plantago asiatica L. by response surface methodology. Carbohydr. Polym..

[49] Zhao C.N., Tang G.Y., Cao S.Y., Xu X.Y., Gan R.Y., Liu Q., Mao Q.Q., Shang A., Li H.B. (2019). Phenolic profiles and antioxidant activities of 30 tea infusions from green, black, oolong, white, yellow and dark teas. Antioxidants.

[50] Pastoriza S., Pérez-Burillo S., Rufián-Henares J. (2017). Ángel How brewing parameters affect the healthy profile of tea. Curr. Opin. Food Sci..

[51] Xi J., Wang B.S. (2013). Optimization of ultrahigh-pressure extraction of polyphenolic antioxidants from green tea by response surface methodology. Food Bioprocess Technol..

[52] Dudonné S., Vitrac X., Coutière P., Woillez M., Mérillon J.-M. (2009). Comparative Study of Antioxidant Properties and Total Phenolic Content of 30 Plant Extracts of Industrial Interest Using DPPH, ABTS, FRAP, SOD, and ORAC Assays. J. Agric. Food Chem..

[53] Li A.-N., Li S., Li H.-B., Xu D.-P., Xu X.-R., Chen F. (2014). Total phenolic contents and antioxidant capacities of 51 edible and wild flowers. J. Funct. Foods.

[54] Tang G.-Y., Zhao C.-N., Xu X.-Y., Gan R.-Y., Cao S.-Y., Liu Q., Shang A., Mao Q.-Q., Li H.-B. (2019). Phytochemical Composition and Antioxidant Capacity of 30 Chinese Teas. Antioxidants.

[55] Costa A.M., Ribeiro W.X., Kato E., Monteiro A.R.G., Peralta R.M. (2008). Production of tannase by *Aspergillus tamarii* in submerged cultures. Braz. Arch. Biol. Technol..

[56] Klibanov A.M., Klibanov A.M., Klibanov A.M. (2001). Improving enzymes by using them in organic solvents. Nature.

[57] Mahapatra K., Nanda R.K., Bag S.S., Banerjee R., Pandey A., Szakacs G. (2005). Purification, characterization and some studies on secondary structure of tannase from *Aspergillus awamori* nakazawa. Process Biochem..

[58] Batra A., Saxena R. (2005). Potential tannase producers from the genera Aspergillus and Penicillium. Process Biochem..

[59] Chen H., Zhou X., Zhang J. (2014). Optimization of enzyme assisted extraction of polysaccharides from *Astragalus membranaceus*. Carbohydr. Polym..

[60] Swer T.L., Mukhim C., Bashir K., Chauhan K. (2018). Optimization of enzyme aided extraction of anthocyanins from *Prunus nepalensis* L. LWT.

